# An Fc-modified monoclonal antibody as novel treatment option for pancreatic cancer

**DOI:** 10.3389/fimmu.2024.1343929

**Published:** 2024-01-22

**Authors:** Martina S. Lutz, Kevin Wang, Gundram Jung, Helmut R. Salih, Ilona Hagelstein

**Affiliations:** ^1^ Clinical Collaboration Unit Translational Immunology, German Cancer Consortium (DKTK), Department of Internal Medicine, University Hospital Tuebingen, Tuebingen, Germany; ^2^ Cluster of Excellence iFIT (EXC 2180) “Image-Guided and Functionally Instructed Tumor Therapies”, University of Tuebingen, Tuebingen, Germany; ^3^ Department of Immunology, Eberhard Karls Universität Tübingen, Tuebingen, Germany

**Keywords:** pancreatic cancer, B7-H3, NK cells, therapeutic antibody, immunotherapy

## Abstract

Pancreatic cancer is a highly lethal disease with limited treatment options. Hence, there is a considerable medical need for novel treatment strategies. Monoclonal antibodies (mAbs) have significantly improved cancer therapy, primarily due to their ability to stimulate antibody-dependent cellular cytotoxicity (ADCC), which plays a crucial role in their therapeutic efficacy. As a result, significant effort has been focused on improving this critical function by engineering mAbs with Fc regions that have increased affinity for the Fc receptor CD16 expressed on natural killer (NK) cells, the major cell population that mediates ADCC in humans. Here we report on the preclinical characterization of a mAb directed to the target antigen B7-H3 (CD276) containing an Fc part with the amino acid substitutions S239D/I332E to increase affinity for CD16 (B7-H3-SDIE) for the treatment of pancreatic cancer. B7-H3 (CD276) is highly expressed in many tumor entities, whereas expression on healthy tissues is more limited. Our findings confirm high expression of B7-H3 on pancreatic cancer cells. Furthermore, our study shows that B7-H3-SDIE effectively activates NK cells against pancreatic cancer cells in an antigen-dependent manner, as demonstrated by the analysis of NK cell activation, degranulation and cytokine release. The activation of NK cells resulted in significant tumor cell lysis in both short-term and long-term cytotoxicity assays. In conclusion, B7-H3-SDIE constitutes a promising agent for the treatment of pancreatic cancer.

## Introduction

Pancreatic cancer is a highly aggressive malignancy with a notably unfavorable overall prognosis and a mortality rate nearly equivalent to its incidence rate. Surgical resection is currently the only curative treatment for this disease, but less than 20% of diagnosed patients are eligible for this procedure (1). For patients with advanced disease, systemic chemotherapy is typically used as first-line therapy, with response rates as low as 32% (2–5). Targeted therapies have also improved treatment options only modestly. The combination of gemcitabine and the tyrosine kinase inhibitor erlotinib showed a slight survival benefit in an unselected patient population (6). Patients with microsatellite instability were the only ones to show clinical benefit from immunotherapy with checkpoint inhibitors, the small molecule inhibitor olaparib was approved for patients with BRCA mutations only (7, 8). This highlights the urgent medical need for new therapeutic options in this patient population.

Immunotherapy with monoclonal antibodies (mAbs) has significantly improved the therapeutic options for several malignant diseases. The therapeutic efficacy of antitumor mAbs is largely attributed to their ability to induce antibody-dependent cellular cytotoxicity (ADCC). However, despite their unquestionable success, the therapeutic efficacy of antitumor mAbs still leaves room for improvement. While several factors can impact the susceptibility of tumor cells to therapeutic antibodies, one potential approach to improve efficacy is to enhance their affinity to the activating Fc receptor CD16 (9). This enhances the recruitment of immune cells that express CD16, among which, at least in humans, natural killer (NK) cells play a significant role (10). Affinity to CD16 can be improved by modifying the Fc parts either with regard to their glycosylation patterns or by changes in the amino acid sequence (11, 12). One frequently pursued modification is the amino acid substitution S239D/I332E (SDIE) that increases the Fc-part’s affinity to FcγR in general, but has a more pronounced effect on the activating FcγRIIIa/CD16a compared to the inhibitory FcγRIIb/CD32b (11).

Limited access of the tumor site for therapeutically stimulated effector cells is a major obstacle for immunotherapy of solid tumors. The type I transmembrane protein B7-H3 (CD276) is overexpressed on a variety of cancers making it a promising target for antibody-based immunotherapeutic approaches (13–17). Robust expression of B7-H3 was reported in the majority of investigated human pancreatic cancer tissues, with notably higher levels observed in comparison to normal pancreatic and non-cancerous tissues (17, 18). Notably, expression exhibited significantly greater intensity in cases featuring lymph node metastasis and advanced pathological stages (17). In addition, B7-H3 is expressed not only on tumor cells, but also on tumor vessels, which may facilitate access of immune effector cells to solid tumors upon therapeutic targeting (19, 20).

In the present study, we set out to characterize the suitability of targeting B7-H3 with a novel Fc-optimized mAb termed B7-H3-SDIE as immunotherapeutic treatment option for pancreatic cancer.

## Materials and methods

### Production and purification of antibodies

The B7-H3-targeting mAb with a wildtype Fc portion B7-H3-WT, the B7-H3-SDIE and the corresponding control iso-SDIE were generated by chimerizing an anti-B7-H3 mAb (clone 8H8) and a control mAb (clone MOPC21), respectively, with the human immunoglobulin G1/K constant region (21). The mAbs were Fc-optimized with S239D/I332E modification according to previous descriptions (22). In brief, the mAb light and heavy chain plasmids were obtained using the EndoFree Plasmid Maxi kit from Qiagen (Hilden, Germany), following the manufacturer’s instructions. Antibodies were produced using the ExpiCHO cell system (Gibco, in Carlsbad, CA) following the manufacturer’s instructions. Antibodies were purified from media by protein A affinity chromatography (GE Healthcare, Chicago, IL) followed by preparative size exclusion chromatography (HiLoad 16/60 Superdex 200; GE Healthcare). To guarantee the quality and purity of the antibodies produced, we conducted analytical size exclusion chromatography (using Superdex 200 Increase 10/300 GL from GE Healthcare) and 4–12% gradient SDS-PAGE gels (Invitrogen, Carlsbad, CA) with gel filtration and Precision Plus standard from Bio-Rad (Hercules, CA), respectively.

Endotoxin levels of samples as determined by an EndoZyme assay (Biomerieux, Nuertingen, Germany) were<0.5 EU/mL.

### Relative gene expression of CD276 based on TCGA database analysis

Relative expression levels of B7-H3 mRNA were obtained for tumor and normal tissue samples from the Cancer Genome Atlas (TCGA) database and the GTEx project, using the Gene Expression Profiling Interactive Analysis (GEPIA) web server as previously described (23). Data sets for pancreatic adenocarcinoma (tumor = 179) and corresponding normal tissue (normal = 171) were downloaded from TCGA (http://www.oncolnc.org). The online web server GEPIA (http://gepia.cancer-pku.cn) was utilized for analysis of B7-H3 RNA levels depending on tumor stage (I-IV).

### Polymerase chain reaction

To determine B7-H3 mRNA, B7-H3 primers were QuantiTect Primer Assay Hs_CD276_1_SG (Qiagen), while RPL13 (Hs_RPL13_1_SG, Qiagen) served as housekeeping gene. For total RNA isolation, 1-2 x 10^6^ cells were used and the High Pure RNA Isolation Kit (Roche, Basel, Switzerland) was employed, followed by cDNA synthesis with FastGeneScriptase II (NIPPON Genetics Europe, Dueren, Germany) in accordance with the manufacturer’s instructions. Reverse transcription-polymerase chain reaction (RT-PCR) was conducted following previously described methods (24, 25). Quantitative PCR (qPCR) was performed utilizing the PerfeCTa SYBR Green FastMix (Quanta Biosciences, Beverly, MA) with a LightCycler 480 instrument from Roche.

### Cells

All cell lines were purchased from ATCC (American Type Culture Collection) and cultured in Dulbeccos Modified Eagle Medium (DMEM) (Gibco). Cells were tested routinely for mycoplasma contamination every three months. Authenticity was determined on a regular basis by validating the respective immunophenotype described by the provider using flow cytometry. Healthy donor peripheral blood mononuclear cells (PBMC) were isolated using density gradient centrifugation (Biochrom, Berlin, Germany). PBMC were collected from healthy donors of various ages and genders, and randomly selected for each experiment. Cryopreserved cells were cultured in media overnight at 37°C prior to use in functional experiments. In every case, written consent was obtained in accordance with the Helsinki protocol. The study was conducted in adherence to the local ethics committee’s guidelines.

### Flow cytometry

Fluorescence-conjugated B7-H3 mAb or isotype control (BioLegend, San Diego, CA) was used to analyze surface expression of B7-H3. To conduct dose titration and binding experiments, cells were stained with B7-H3-SDIE or iso-SDIE, followed by an anti-human PE conjugate (Jackson ImmunoResearch West Grove, PA). A murine B7-H3 hybridoma-derived antibody and the QIFIKIT (Dako, Hamburg, Germany) were employed to perform a quantitative analysis of immunofluorescence aimed to determine the number of B7-H3 molecules present on the cell surface, as previously described (22).

For staining of NK cell activation and degranulation, fluorescence-labeled CD3-APC/Fire, CD56-PECy, CD16-APC, CD25-BV711, CD69-PE (all from BioLegend), or CD107a-PE (BD Biosciences, Franklin Lakes, NJ) were used to stain the cells.

Target cell lysis was assessed *via* flow cytometry using a previously described method (26). In brief, pancreatic cancer cells were seeded in coculture with PBMC from healthy donors, with or without antibodies (1 μg/mL each), after loading with CellTrace™ Violet cell proliferation dye (Thermo Fisher Scientific, Waltham, MA) at 2.5 μM. To control for assay volumes, beads (Sigma, St. Louis, MO) were used. The percentage of viable target cells was determined by calculating the proportion of 7-AAD-negative cells after treatment relative to those in the control group and multiplying by 100.

The exclusion of dead cells from flow cytometric analysis was achieved using 7-AAD (BioLegend) staining (1:200) or LIVE/DEAD™ Fixable Aqua (Thermo Fisher Scientific). The BD FACS Canto II or BD Fortessa (BD Biosciences) were used for sample analysis. Data analysis was carried out with FlowJo software (FlowJo LCC, Ashland, OR).

### NK cell activation and degranulation assays

To assess the activation and degranulation of NK cells in healthy donor PBMC, coculture experiments were conducted with 20,000 pancreatic cancer cells and PBMC at an effector-to-target (E:T) ratio of 2.5:1 in the absence or presence of treatment (1 μg/mL). For degranulation analysis, Brefeldin A (GolgiPlug, BD Biosciences) was added to the coculture and cells were harvested after 4 hours. Subsequently, cells were stained for CD107a expression and analyzed using flow cytometry. After 24 hours, the cells were collected and labeled to evaluate CD69 and CD25 expression by flow cytometric analysis. NK cells were identified as CD3^-^ CD56^+^ cells within PBMC.

### Analysis of cytokine secretion

For the analysis of cytokine secretion, NK cells or PBMC from healthy donors were cultured with or without 20,000 pancreatic cancer cells at an E:T ratio of 2.5:1 in the presence or absence of the proposed mAbs at a concentration of 1 µg/mL. After a 4-hour incubation, the supernatants from the coculture were analyzed for the secretion of IFN-γ and TNF using Legendplex assays (BioLegend) following the manufacturer’s recommendations.

### Cytotoxicity assays

The cytotoxicity of PBMC against pancreatic cancer cells was evaluated using BATDA Europium assays (PerkinElmer, Waltham, MA) over a 2-hour duration, as previously described (27). The percentage of specific lysis was calculated using the formula:


100×[(experimental release)−(spontaneous release)]/[(maximum release)−(spontaneous release)].

To conduct long-term cytotoxicity analyses, we employed the xCELLigence RTCA system (Roche Applied Science, Penzberg, Germany) to perform a real-time assay at 30-minute intervals for a 120-hour observation period. Pancreatic cancer cells (5,000 cells/well) were seeded in 96-well plates and cocultured with PBMC from healthy donors at an E:T ratio of 10:1; this was done with and without the specified mAbs (1 µg/mL each).

### Statistics

Statistical analyses were conducted using GraphPad Prism software (version 9) (GraphPad, Boston, MA). Mean ± standard deviation of replicates or individual values were used to present data. Continuous variables were analyzed using Student’s t-test, Mann-Whitney U test, one-way ANOVA, and Friedman’s test. In the case of significant differences observed by ANOVA, group-wise comparison was performed using Tukey’s multiple comparison test. In the case of significant differences found by Friedman’s test, Dunn’s multiple comparison test was employed. All statistical tests were considered statistically significant when *p* was below 0.05.

## Results

### B7-H3 expression and binding of B7-H3-SDIE antibody in pancreatic cancer cells

**Figure 1:**
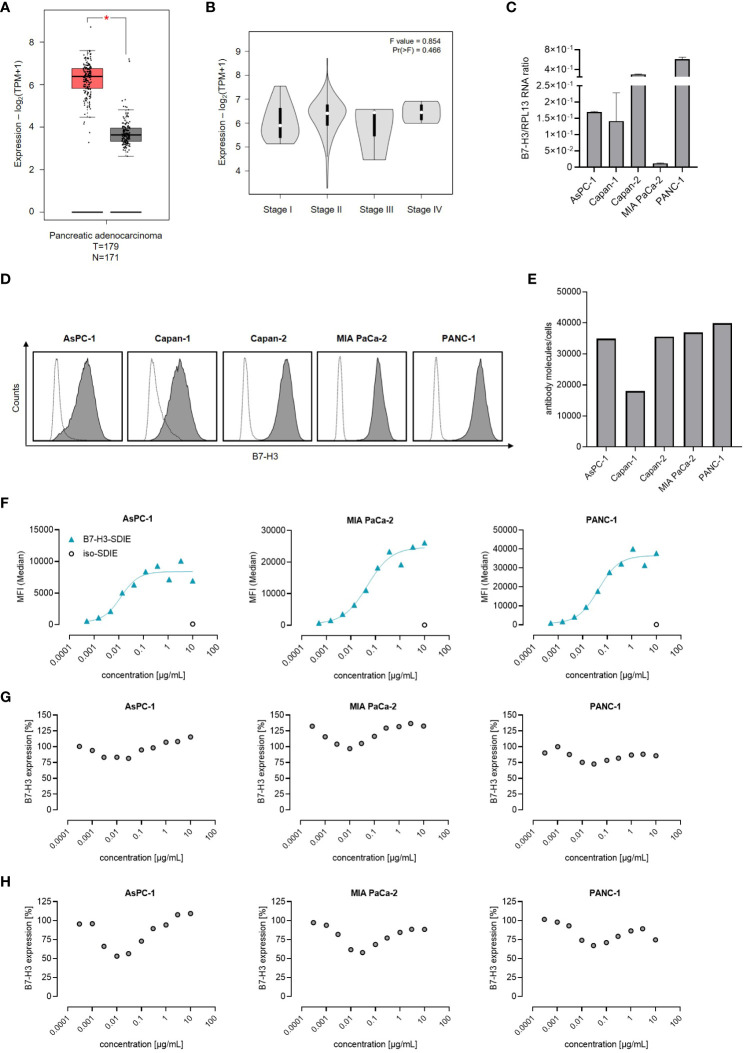
B7-H3 Expression and antibody binding in pancreatic cancer cells. **(A, B)** The mRNA expression of B7-H3 was evaluated through the use of the online tool GEPIA in **(A)** tumor (T) and corresponding normal (N) tissues, as well as in B pancreatic cancer cases at various disease stages. **(C)** Analysis of B7-H3 mRNA expression compared to RPL13 mRNA in five pancreatic cancer cell lines. Combined results are shown for three independent experiments. **(D)** B7-H3 surface expression on specified pancreatic cancer cells was examined *via* flow cytometry using mAb against B7-H3 (shaded peaks) and an isotype control (open peaks). Exemplary histograms from one representative experiment of a total of three with similar results are provided. **(E)** B7-H3 molecules were quantified in pancreatic cancer cell lines using FACS. Results from two independent experiments are presented. **(F)** Pancreatic carcinoma cell lines were treated with B7-H3-SDIE or iso-SDIE at the indicated concentrations, followed by analysis with anti-human PE conjugate using flow cytometry. Data for mean fluorescence intensity (MFI) levels from a representative experiment of three with similar results are displayed. **(G, H)** Pancreatic cancer cells AsPC-1, MIA PaCa-2, and PANC-1 were incubated with B7-H3-SDIE at specified concentrations or iso-SDIE (10 µg/mL) for **(G)** 24 hours and **(H)** 72 hours, respectively. The cells were subsequently washed and reincubated with 1 μg/mL of B7-H3-SDIE, followed by an anti-human PE conjugate (1:200), and analyzed using flow cytometry. The relative surface expression of B7-H3 was determined by defining the MFI of cells preincubated without an antibody as 100%. Exemplary data from one representative experiment out of a total of three is presented. The results depict mean ± SD.

B7-H3 expression has been reported in multiple solid tumors, including pancreatic cancer (28, 29). As a first step, we analyzed B7-H3 mRNA expression using TCGA datasets consisting of 179 pancreatic adenocarcinomas and 171 normal pancreatic tissue samples. Our analysis reveals that B7-H3 mRNA expression is substantially higher in pancreatic cancer tissues than in normal tissues (**Figure 1A**). A comparative analysis of B7-H3 mRNA levels in the TCGA data set of pancreatic adenocarcinomas, categorized by disease stage (I-IV), showed no significant differences in B7-H3 expression levels (**Figure 1B**). Next we studied B7-H3 expression mRNA levels in the pancreatic cancer cell lines AsPC-1, Capan-1, Capan-2, MIA PaCa-2, and PANC-1 (30), revealing differing B7-H3 expression levels across the examined cell lines (**Figure 1C**). A key determinant for an effective immunotherapeutic target antigen is its expression level on the cell surface. Our analysis, conducted by flow cytometry, revealed that B7-H3 is expressed on the cell surface of all pancreatic cancer cell lines tested, albeit to varying extent (**Figure 1D**). The range of B7-H3 surface molecules varied from 18,030 (Capan-1) to 39,840 molecules/cell (PANC-1) (**Figure 1E**). These results led us to select AsPC-1, MIA PaCa-2, and PANC-1 for functional assessments implementing our B7-H3 targeting Fc-optimized B7-H3-SDIE mAb. Dose titration experiments (ranging from 0.0005-10 µg/mL) showed that binding of B7-H3-SDIE saturates at a concentration of 1 µg/mL (**Figure 1F**). This concentration was used for subsequent functional analyses. Binding of an antibody to its target molecule frequently results in dose-dependent modulation of target antigen expression levels. This, in turn, may impair therapeutic efficacy of mAb treatment (31). To assess the induction of this phenomenon known as antigen shift, we examined B7-H3 expression after incubating pancreatic cancer cells with B7-H3-SDIE (range 0.0003-10 µg/mL) for 24 and 72 h. We observed only a slight decrease (maximum of 25% reduction) in B7-H3 surface expression (**Figures 1G, H**).

### Effects of B7-H3-SDIE in the absence of B7-H3^+^ tumor cells

**Figure 2:**
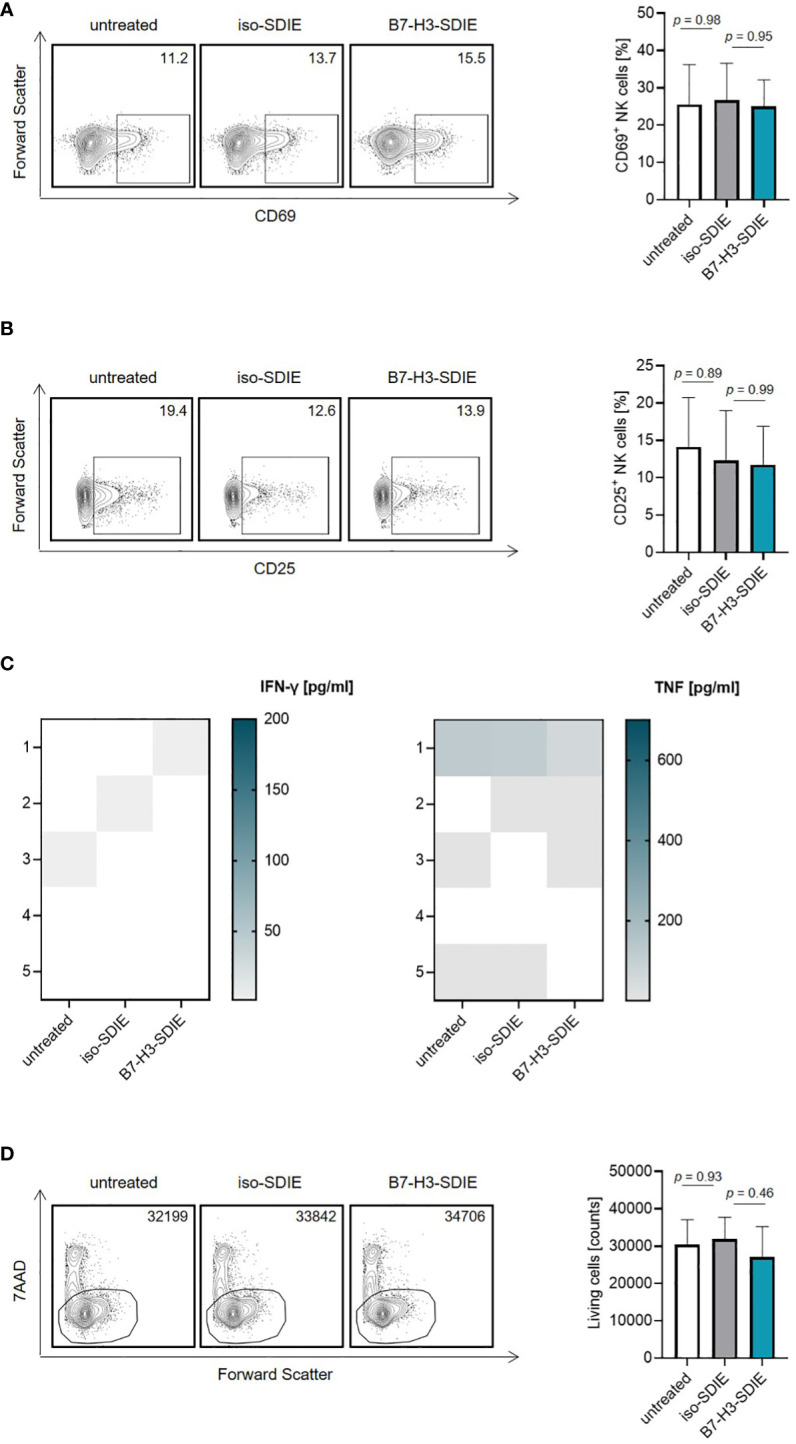
Effects of B7-H3-SDIE in the absence of B7-H3^+^ tumor cells. Effector cells of healthy donors were cultured without target cells in the presence or absence of soluble B7-H3-SDIE or the corresponding isotype control (1 μg/mL). **(A, B)** Activation of NK cells was determined by expression of **(A)** CD69 and **(B)** CD25 after 24 h. In the left panels, exemplary flow cytometry results obtained with one donor and in the right panel combined data with NK cells of n = 5 different donors are shown. **(C)** Cytokine release was analyzed using Legendplex assays for supernatants of cocultures after 4 hours. **(D)** Total numbers of living PBMC were analyzed by flow cytometry after 24 h. In the left panels, exemplary flow cytometry results for living cell counts obtained with one PBMC donor and in the right panel data with PBMC of n = 5 different donors are shown. Results are shown as mean ± SD.

Next, we investigated the effects of B7-H3-SDIE on NK cells in the absence of tumor cells. To this end, isolated NK cells or whole PBMC were incubated with B7-H3-SDIE or corresponding isotype control. B7-H3-SDIE did not induce NK cell activation (**Figures 2A, B**) or secretion of the immune effector cytokines IFN-γ and TNF (**Figure 2C**) in the absence of target cells. Furthermore, we did not observe any changes of total PBMC numbers, confirming that B7-H3-SDIE does not induce unspecific cell lysis (**Figure 2D**).

### Induction of NK cell reactivity by B7-H3-SDIE against pancreatic cancer cells

**Figure 3:**
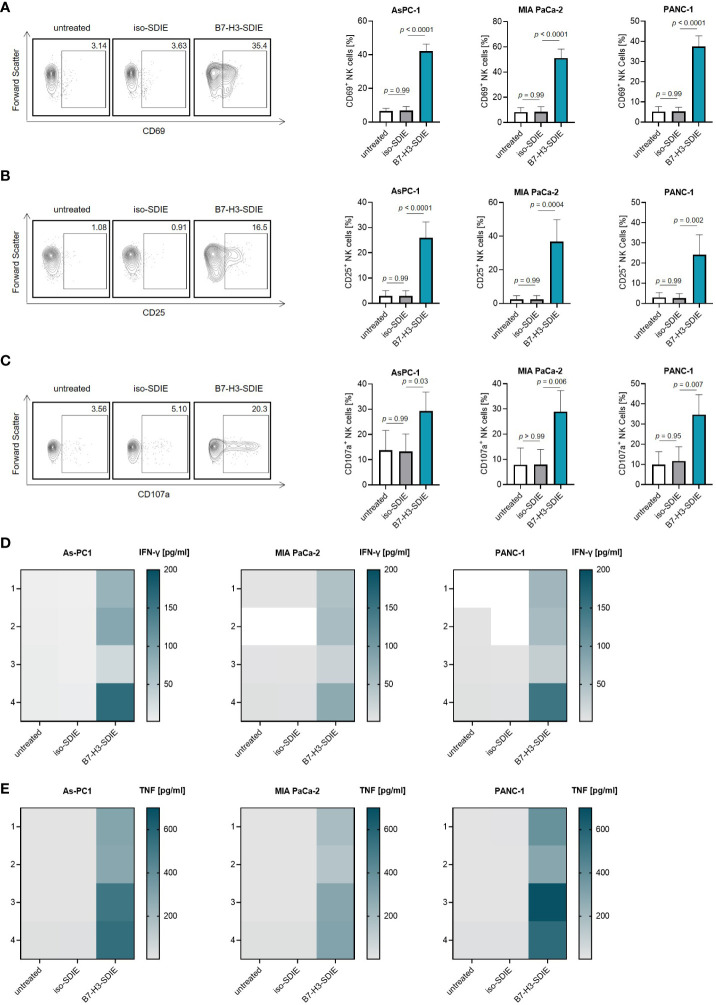
Induction of NK cell reactivity by B7-H3-SDIE against pancreatic cancer cells. PBMC from healthy donors were cultured with pancreatic cancer cells including AsPC-1, MIA PaCa-2, and PANC-1 at 2.5:1 E:T ratio with or without the B7-H3-SDIE or corresponding isotype control (1 μg/mL). **(A, B)** Activation of NK cells was assessed by analyzing the expression of **(A)** CD69 and **(B)** CD25 after 24 hours. The left panels show results of flow cytometry testing conducted on a single PBMC donor along with PANC-1 cells. The right panels present data on pancreatic cancer cell lines with PBMC from four diverse donors. **(C)** NK cell degranulation was assessed by CD107a expression after 4 hours. The left panels display flow cytometry data obtained from one PBMC donor, while the right panels exhibit results from four independent PBMC donors with pancreatic cancer cell lines. **(D, E)** Cytokine release was analyzed using Legendplex assays for supernatants of cocultures after 4 hours. The release of **(D)** IFNγ and **(E)** TNF is presented for n = 4 PBMC donors, respectively. Mean ± SD is shown for the results.

As a next step, we investigated if and how our mAb B7-H3-SDIE activates immune effector cells to attack pancreatic cancer cells. For this purpose, we used PBMC from healthy donors as effector cells. Cocultures of PBMC and pancreatic cancer cells (AsPC-1, MIA PaCa-2, and PANC-1) in the presence or absence of B7-H3-SDIE or control mAb iso-SDIE were analyzed for the expression of the activation markers CD69 and CD25 on NK cells upon counterstaining for CD56, CD3 and CD16 after 24 h by flow cytometry. A comparison between the Fc-optimized B7-H3-SDIE and a Fc wildtype mAb (B7-H3-WT) revealed that B7-H3-SDIE binds more strongly to CD16 on NK cells, resulting in increased activation capacity (**Supplementary Figures S1A-C**). Both CD16^+^ and CD16^-^ NK cells showed greater activation when exposed to B7-H3-SDIE compared to B7-H3-WT or control treatments (**Supplementary Figures S1D, E**). Following treatment with B7-H3-SDIE, the proportion of CD16^+^ NK cells decreased significantly compared to treatment with B7-H3-WT (**Supplementary Figure S1F**). Following treatment with B7-H3-SDIE, the activation markers CD69 and CD25 were significantly upregulated on NK cells. The control mAb iso-SDIE with nonrelevant target specificity had no significant effect (**Figures 3A, B**). B7-H3-SDIE induced activation of various NK cell subsets (CD56^bright^CD16^-^, CD56^dim^CD16^-^ and CD56^dim^CD16^+^) with most pronounced effects observed with the CD56^dim^CD16^+^ subpopulation (**Supplementary Figure S2**). This subpopulation is crucial for treatment with ADCC inducing mAbs, as it is not only the major circulating subset, but also the primary ADCC inducing subset (32, 33). Flow cytometric analysis further showed increased expression of the degranulation marker CD107a on NK cells in cocultures of PBMC with pancreatic carcinoma cells when treated with B7-H3-SDIE. In contrast, the Fc control mAb iso-SDIE had no significant effect (**Figure 3C**). In line, degranulation was induced in all analyzed NK cell subsets (CD56^bright^CD16^-^, CD56^dim^CD16^-^ and CD56^dim^CD16^+^) (**Supplementary Figure S3**). Activated NK cells produce cytokines, such as IFN-γ and TNF, that can effectively stimulate an immune response to fight tumors. These cytokines can directly impact target cells or activate other immune cells to attack target cells (34). Legendplex assays used to analyze cytokine secretion into culture supernatants of PBMC and pancreatic cancer cell lines demonstrated a significant increase in the release of both IFN-γ and TNF after treatment with B7-H3-SDIE, whereas the isotype control again had no relevant effect (**Figures 3D, E**).

### Induction of target cell lysis by Fc-optimized B7-H3-SDIE antibody

**Figure 4:**
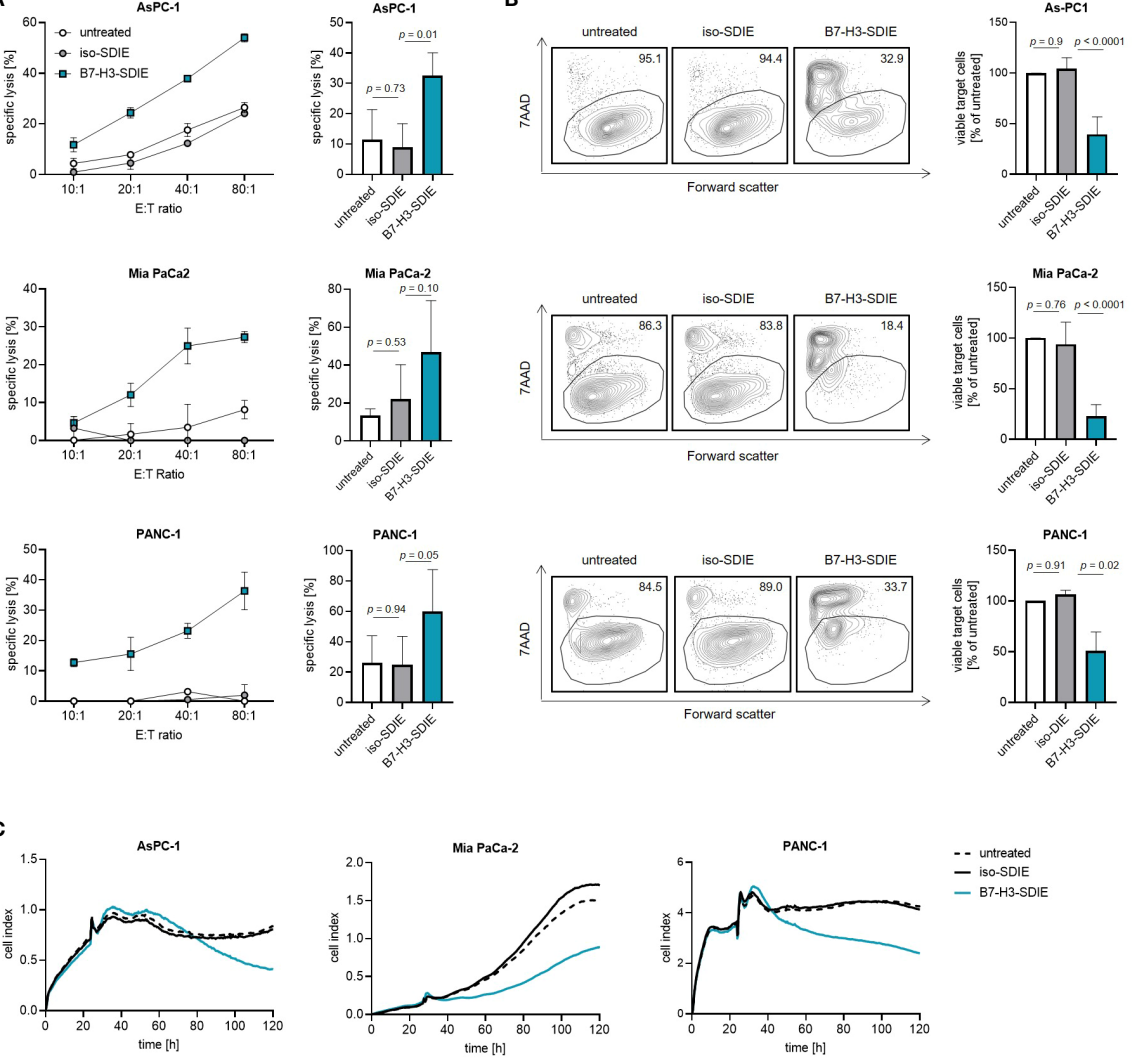
Induction of target cell lysis by Fc-optimized B7-H3-SDIE antibody. PBMC from healthy donors were incubated with AsPC-1, MIA PaCa-2, and PANC-1 pancreatic cancer cells and treated with B7-H3-SDIE or control antibody iso-SDIE (1 μg/mL). **(A)** Lysis of pancreatic cancer cell lines was analyzed using 2-hour Europium cytotoxicity assays. Exemplary data from a single PBMC donor at various E:T ratios are presented in the left panels, while pooled data from PBMC donors (n=3-5) at an E:T ratio of 40:1 are displayed in the right panels. **(B)** Lysis of pancreatic cancer cell lines was evaluated using flow cytometry-based lysis assays after 72 hours, with an E:T ratio of 10:1. The left panels depict exemplary dot plots featuring one PBMC donor, while the right panel displays the combined results for each cell line with n=3-5 PBMC. **(C)** Using xCELLigence, cell death of pancreatic cancer cells was determined. PBMC were incubated with target cells at an E:T ratio of 10:1 for 120 hours. Exemplary data from a representative experiment of three total experiments are shown. Results are presented as mean ± SD.

Finally, to study the ability of B7-H3-SDIE to induce tumor cell lysis, we co-cultured the pancreatic cancer cell lines AsPC-1, MIA PaCa-2, and PANC-1 with PBMC from healthy donors. We found that B7-H3-SDIE substantially induced lysis of target cells in short-term cytotoxicity assays for all the cell lines examined, whereas the presence of control mAb exhibited no discernible impact on target cell lysis (**Figure 4A**). Analyses using long-term flow cytometry-based lysis assays over 72 h further demonstrated the marked efficacy of B7-H3-SDIE against pancreatic cancer cells (**Figure 4B**). The potential of B7-H3-SDIE to induce target cell lysis was additionally confirmed by analyzing tumor cell lysis over a 120-hour period using xCELLigence assays, despite the varying morphological features, growth rates, and levels of B7-H3 surface expression across the different pancreatic cancer cell lines (**Figure 4C**). To summarize, our findings have demonstrated that B7-H3-SDIE exerts a significant effect on the activation, degranulation, and cytokine release of NK cells, ultimately resulting in the lysis of tumor cells.

## Discussion

Although advances in detection and treatment have led to declining death rates for certain types of cancer, the overall mortality rate for pancreatic cancer remains high (35, 36). Due to the stagnation of progress in treatment in recent years, it is anticipated that pancreatic cancer will become the second highest cause of cancer-related death worldwide by 2030 (37, 38). Accordingly, new therapeutic concepts are urgently needed. In the present study, we report on the preclinical characterization of an Fc-optimized mAb targeting B7-H3 for induction of NK cell reactivity against pancreatic cancer cells.

NK cells play a critical role as effector cells in cancer immunosurveillance. Accordingly, NK cells have received considerable attention for immunotherapies, including both cellular and pharmaceutical approaches. Interestingly, studies in pancreatic cancer showed that NK cell frequencies are increased and also the NK phenotype was altered after duodenopancreatectomy (39, 40). However, the precise role of NK cells in pancreatic cancer remains unclear, but an increasing body of evidence underscores the significance of NK cells in this disease. Studies analyzing the phenotype and function of NK cells in the blood and pancreatic tumors reported a higher percentage of NK cells in the blood of pancreatic cancer patients compared to healthy donors, but the frequency of NK cells in pancreatic tumors is very low (<0.5%). This may be due to the absence of the chemokine receptor CXCR2 on patients’ NK cells, resulting in reduced trafficking of the cells into the tumor (41, 42). Furthermore, NK cells were shown to have difficulty in killing autologous pancreatic cancer cells due to inadequate ligation of the activating NK cell receptors NKG2D and DNAM-1. These issues were resolved by ex-vivo stimulation of NK cells from patients (41). Based on this, it is likely that the use of antitumor mAbs could address both of these limitations.

Since the induction of ADCC is one of the major effector mechanisms mediated by antitumor mAbs, many efforts are currently aimed at reinforcing the efficacy of antitumor mAbs by increasing the affinity of the Fc part to CD16 that is expressed on NK cells, and thus improving ADCC. Besides modifying glycosylation motifs (12), increased affinity to CD16 can be achieved by modifying the amino acid sequence in the CH2 domain of the Fc part, for example by the S239D/I332E substitutions (SDIE modification) (11) which is also contained in our B7-H3-targeting mAb. Discussions regarding the influence of a polymorphism at position 158 on the affinity of FcγRIIIa to IgG1 Fc parts (V, high-affinity receptor; F, low-affinity receptor) have been ongoing (43, 44). Previous studies have analyzed the influence of this polymorphism on the efficacy of SDIE-modified constructs and did not find a significant influence (45). It should also be emphasized that the SDIE modification not only improved ADCC mediated by NK cells, but also by certain subsets of monocytes/macrophages, and additional analyses are warranted to investigate whether and how B7-H3-SDIE affects effector functions of cell populations other than NK cells (46). In previous studies, we evaluated mAbs containing the SDIE modification for treatment of malignancies such as leukemia, colorectal cancer and sarcoma, some of them until the stage of clinical validation (21, 47–49).

B7-H3, also known as CD276, is an immunoregulatory member of the B7 family. Due to its overexpression in tumor tissues including pancreatic cancer while exhibiting limited expression in normal tissues, it constitutes an attractive target for cancer immunotherapy (13, 28). B7-H3 was reported to be overexpressed in 93% of pancreatic cancer tissues. Furthermore, its expression was found to be correlated with lymph node metastasis and a less differentiated tumor grade (50). It has been reported that B7-H3 has a role in regulating pancreatic tumor progression and that inhibition of B7-H3 expression reduced metastasis (14). Moreover, blocking B7-H3 promoted infiltration of CD8^+^ T cells into the tumor and induced substantial anti-tumor effects *in vivo* (17). Due to its elevated expression in the tumor microenvironment and tumor vasculature in various malignancies, B7-H3 has received significant attention as immunotherapeutic target antigen (19, 51). The approach to target the structures surrounding a tumor has the potential to allow enhanced infiltration of immune effector cells into solid tumors, representing a promising strategy for overcoming a significant hurdle to T cell-based immunotherapy. Based on these characteristics, we reasoned that B7-H3 constitutes a promising target for an NK cell activating immunotherapeutic approach for treatment of pancreatic cancer. Initial analysis of TCGA datasets confirmed a significantly higher B7-H3 RNA expression in pancreatic cancer tissue compared to healthy tissue, and flow cytometric analyses confirmed B7-H3 expression on various pancreatic cancer cell lines, albeit to differing extents. Multiple functional *in vitro* assays, including analysis of NK cell activation, degranulation, cytokine release as well cytotoxicity revealed that B7-H3-SDIE is well suited to target pancreatic cancer cells because it potently induced anti-tumor immunity. When we analyzed degranulation in the CD56^bright^CD16^-^, CD56^dim^CD16^-^, and CD56^dim^CD16^+^ NK cell subpopulations, we observed increased degranulation in all NK cell subpopulations. Several studies have shown that blocking/masking B7-H3 using antibodies or B7-H3 knock-down tumor cells increases NK cell cytotoxicity towards B7-H3^+^ target cells (52, 53). Available data document that not only CD56^dim^CD16^+^ but also CD56^bright^CD16^-^ NK cells can acquire cytotoxic capabilities after activation, e.g. in lymph nodes (54). Since the B7-H3-SDIE potently increased cytokine production and effector molecule release compared to mAbs containing the wildtype Fc, the “general immune activation” may thus have induced lytic functions in the CD56^bright^CD16^-^ NK cells. Another explanation would be that the observed effect with seemingly CD16^-^ NK cells is due to the fact that FcR binding results in downregulation of CD16 expression after treatment with B7-H3-SDIE. Finally, it is tempting to speculate that B7-H3-SDIE, beyond inducing Fc-mediated ADCC, may block the effect of B7-H3 which has been reported to inhibit overall NK cell reactivity.

The microenvironment of the tumor is an important factor for therapeutic success when using therapeutic antibodies. This factor is not mirrored in our experimental systems and should be considered in further preclinical investigations.

Several clinical trials are currently underway to investigate various approaches targeting B7-H3. This includes radiolabeled mAbs (131I-omburtamab, 177Lu-DPTA omburtamab), antibody-drug conjugates (MGC018; DS7300a) and Fc-optimized mAbs (MGA271, enoblituzumab; DS-5573a, clinical trial completed) (55, 56). The B7-H3 antibody-drug conjugate (MGC018) is being evaluated in combination with a PD-1xCTLA-4 DART molecule (NCT05293496) for the treatment of advanced solid tumors, including those affecting the pancreas but results are not yet available. Furthermore, a bispecific antibody (MGD009) has been clinically tested. Another approach which is frequently pursued are B7-H3 targeting CAR T cells, which are being evaluated in various tumor entities including advanced pancreatic carcinoma (NCT04897321, NCT05211557, NCT05341492, NCT04483778, NCT05323201, NCT05241392, NCT05474378, NCT03198052, NCT04185038, NCT05366179, NCT04670068, NCT05143151). Furthermore, various B7-H3 targeting approaches are currently under preclinical evaluation like, but not limited to a B7-H3 TriKE (GBT-5550), camel nanobody-based CAR T cells (57), a dual nanobody NK cell engager (58) and bispecific antibodies (59–62).

A major obstacle to antibody-based immunotherapy is the expression of target antigens on healthy tissues and cells, which may result in undesirable side effects. It is known that B7-H3 is expressed at high levels in a variety of tumor types. Conversely, it is either absent or expressed at low levels in normal tissue and cells such as lymphocytes or isolated healthy donor PBMC (53), yet the expression level increases on these cells upon stimulation (63, 64). When considering toxicity of B7-H3, it is important to note that this protein is expressed on antigen-presenting cells, endothelial cells, resting fibroblasts, amniotic fluid stem cells and osteoblasts (63, 65). Low B7-H3 expression was also reported in healthy liver tissue by immunohistochemistry (66). However, preclinical investigations utilizing therapeutic strategies targeting B7-H3, including B7-H3-targeting CAR T cells, have demonstrated a significant anti-tumor effect in experimental models in an absence of associated toxicity, despite the weak expression of B7-H3 on certain non-tumor cells (23, 67, 68). Consistently, we did not observe off-target activity of our B7-H3-SDIE in the absence of tumor cells. In addition, the safety of an mAb is expected to be higher than that of T cell-based approaches, as NK cells release less pro-inflammatory cytokines, which can potentially lead to cytokine release syndrome, compared to T cells (69–71).

Taken together, our study demonstrates that our B7-H3-targeting mAb containing the SDIE modification to enhance ADCC, effectively induces anti-tumor immunity in pancreatic cancer, indicating that B7-H3-SDIE constitutes a promising novel option for pancreatic cancer treatment.

## Data availability statement

The raw data supporting the conclusions of this article will be made available by the authors, without undue reservation.

## Ethics statement

The studies involving humans were approved by IRB (ethics committee of the Faculty of Medicine of the Eberhard Karls Universitaet Tuebingen) at the University Hospital Tuebingen. The studies were conducted in accordance with the local legislation and institutional requirements. The participants provided their written informed consent to participate in this study.

## Author contributions

ML: Conceptualization, Data curation, Formal Analysis, Investigation, Methodology, Visualization, Writing – original draft, Software. KW: Data curation, Formal Analysis, Methodology, Writing – review & editing. GJ: Resources, Supervision, Writing – review & editing. HS: Conceptualization, Funding acquisition, Resources, Supervision, Writing – review & editing. IH: Conceptualization, Investigation, Project administration, Supervision, Validation, Visualization, Writing – original draft.
