# Supplementary material for: An Fc-modified monoclonal antibody as novel treatment option for pancreatic cancer
Source: Front Immunol. 2024 Jan 22;15:1343929. doi: 10.3389/fimmu.2024.1343929 (PMC10845339; doi:10.3389/fimmu.2024.1343929)
Supplement: Supplementary file 1 [file DataSheet_1.pdf]

Supplementary Figures

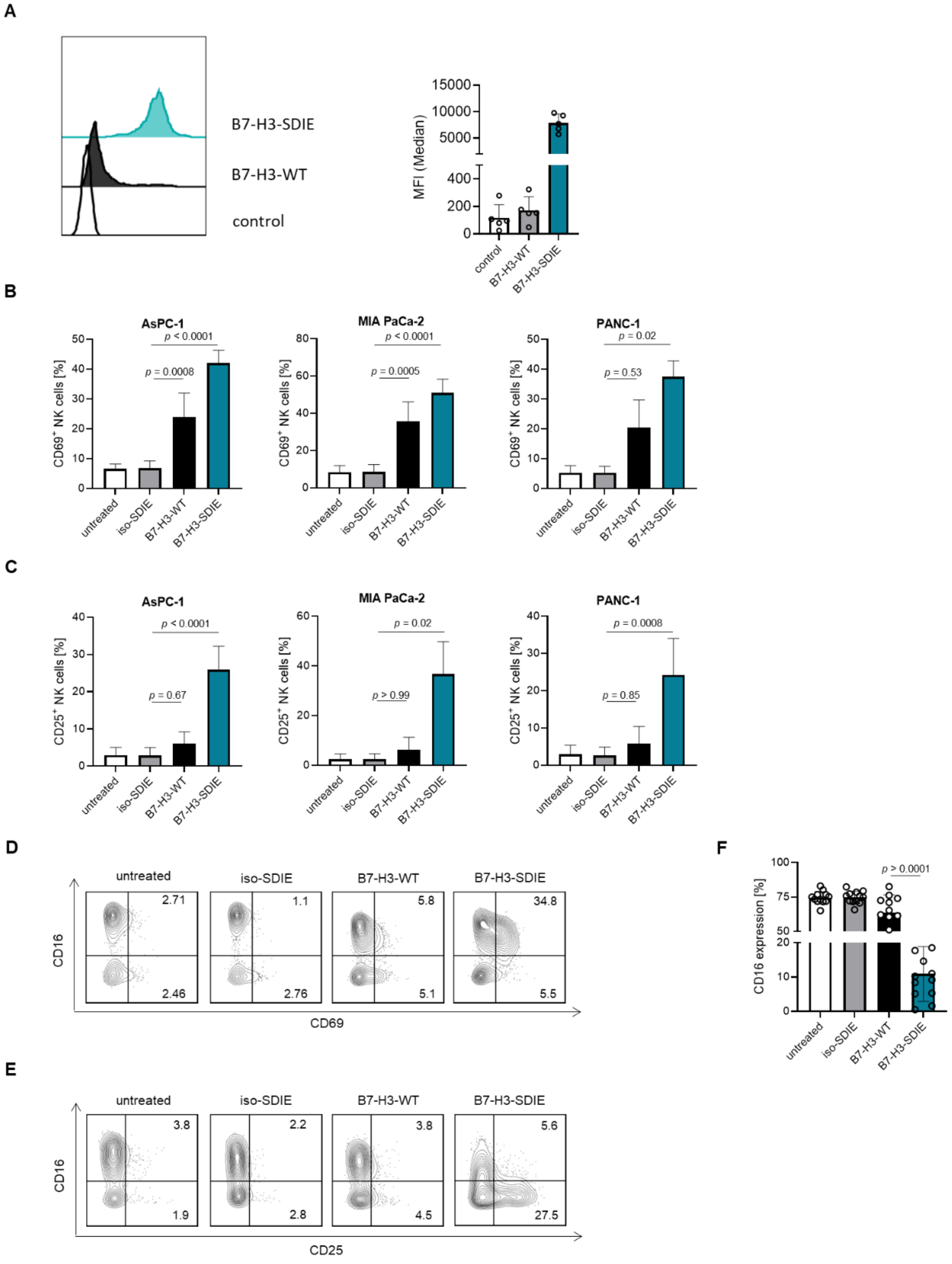

Figure S1: The SDIE-modification enhances binding to CD16 and activation of NK cells. **A** Specific binding of B7-H3 mAbs (1 µg/ml) to CD16 on NK cells was analyzed by flow cytometry using the respective mAbs followed by an anti-human PE conjugate. **B-F** PBMC from healthy donors

(n=4) were cultured with pancreatic cancer cells including AsPC-1, MIA PaCa-2, and PANC-1 at 2.5:1 E:T ratio with or without the B7-H3-SDIE or corresponding isotype control (1 µg/mL) followed by flow cytometry analysis. Activation of NK cells was assessed by analyzing the expression of **B** CD69 and **C** CD25 after 24 hours. **D** Activation was determined depending on CD16 expression of NK cells by analyzing CD69 expression (% of $CD3^-CD56^+$ cells) after 24 hours by flow cytometry. Shown are exemplary dot plots for one cell line with one PBMC donor. **E** Activation was determined depending on CD16 expression of NK cells by analyzing CD25 expression (% of $CD3^-CD56^+$ cells) after 24 hours by flow cytometry. Shown are exemplary dot plots for one cell line with one PBMC donor (not the same donor as shown in Figure S1D). **F** CD16 expression (% of $CD3^-CD56^+$ cells) of NK cells is shown as combined results for all pancreatic cancer cell lines and n = 4 PBMC donors after 24 hours by flow cytometry. Results are shown as mean ± SD.

A

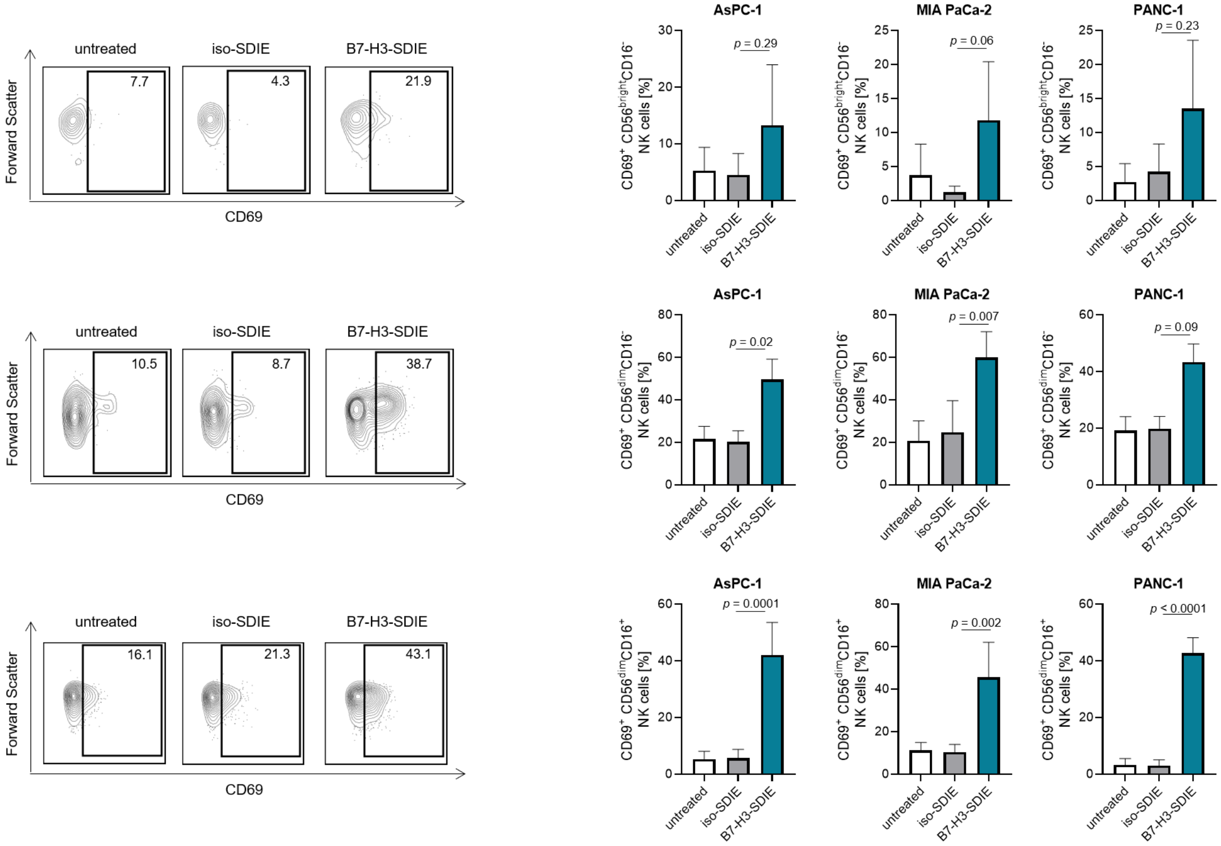
untreated
iso-SDIE
B7-H3-SDIE
Forward Scatter
CD69
7.7
4.3
21.9
10.5
8.7
38.7
16.1
21.3
43.1
AsPC-1
MIA PaCa-2
PANC-1
CD69+ CD56brightCD16- NK cells [%]
CD69+ CD56dimCD16- NK cells [%]
CD69+ CD56dimCD16+ NK cells [%]
p = 0.29
p = 0.06
p = 0.23
p = 0.02
p = 0.007
p = 0.09
p = 0.0001
p = 0.002
p < 0.0001

B

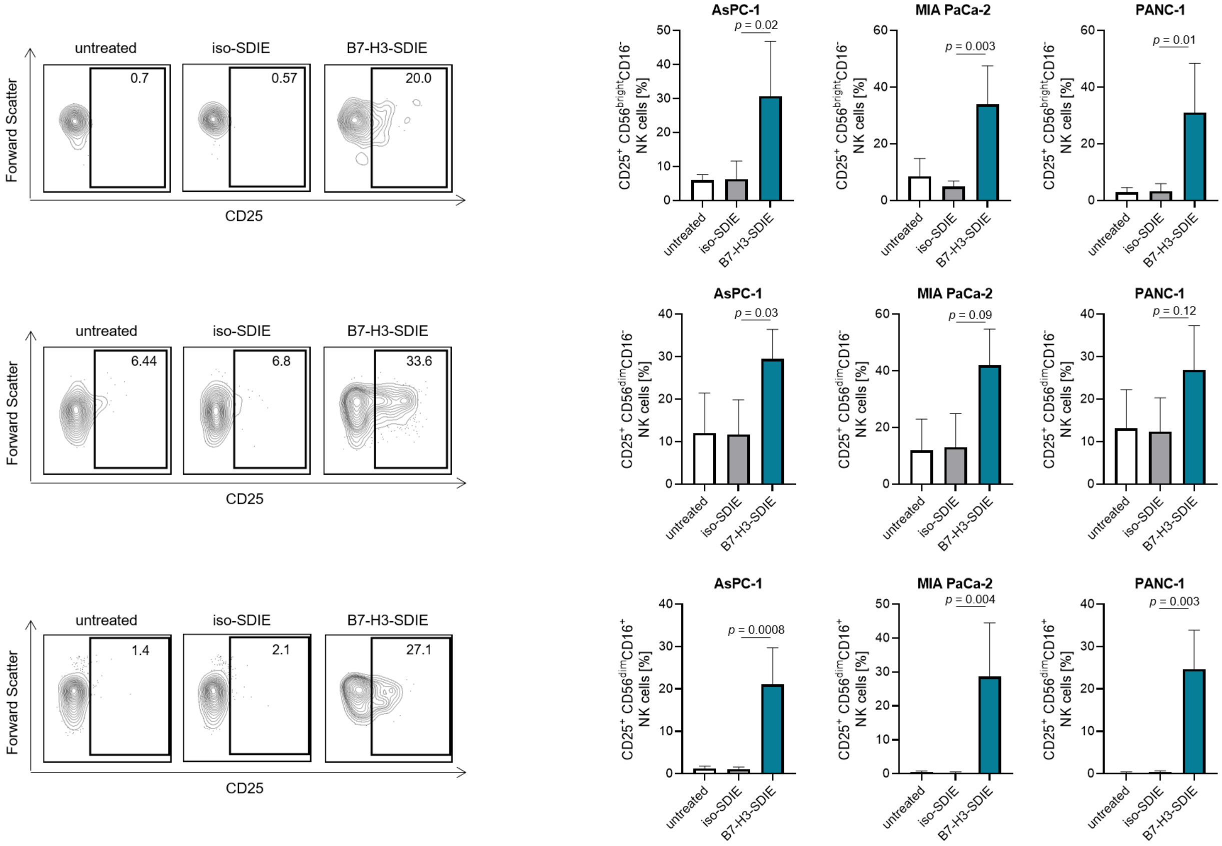
untreated
iso-SDIE
B7-H3-SDIE
Forward Scatter
CD25
0.7
0.57
20.0
6.44
6.8
33.6
1.4
2.1
27.1
AsPC-1
MIA PaCa-2
PANC-1
CD25+ CD56brightCD16- NK cells [%]
CD25+ CD56dimCD16- NK cells [%]
CD25+ CD56dimCD16+ NK cells [%]
p = 0.02
p = 0.003
p = 0.01
p = 0.03
p = 0.09
p = 0.12
p = 0.0008
p = 0.004
p = 0.003

Figure S2: Activation of NK cell subsets (CD56^{bright}CD16^{-}, CD56^{dim}CD16^{-} and CD56^{dim}CD16^{+}) by B7-H3-SDIE. PBMC from healthy donors (n=4) were cultured with pancreatic cancer cells including AsPC-1, MIA PaCa-2, and PANC-1 at an E:T ratio of 2.5:1 with or without the B7-H3-SDIE or corresponding isotype control (1 μg/mL) followed by flow cytometry analysis. Activation of CD56^{bright}CD16^{-}, CD56^{dim}CD16^{-} and CD56^{dim}CD16^{+} NK cells was assessed by analyzing the expression of **A** CD69 and **B** CD25 after 24 hours. On the left, exemplary dot lots for one PBMC donor and one pancreatic cancer cell line, and on the right, combined data for each pancreatic cancer cell line with n = 4 independent PBMC donors are shown. Mean ± SD is shown for the results.

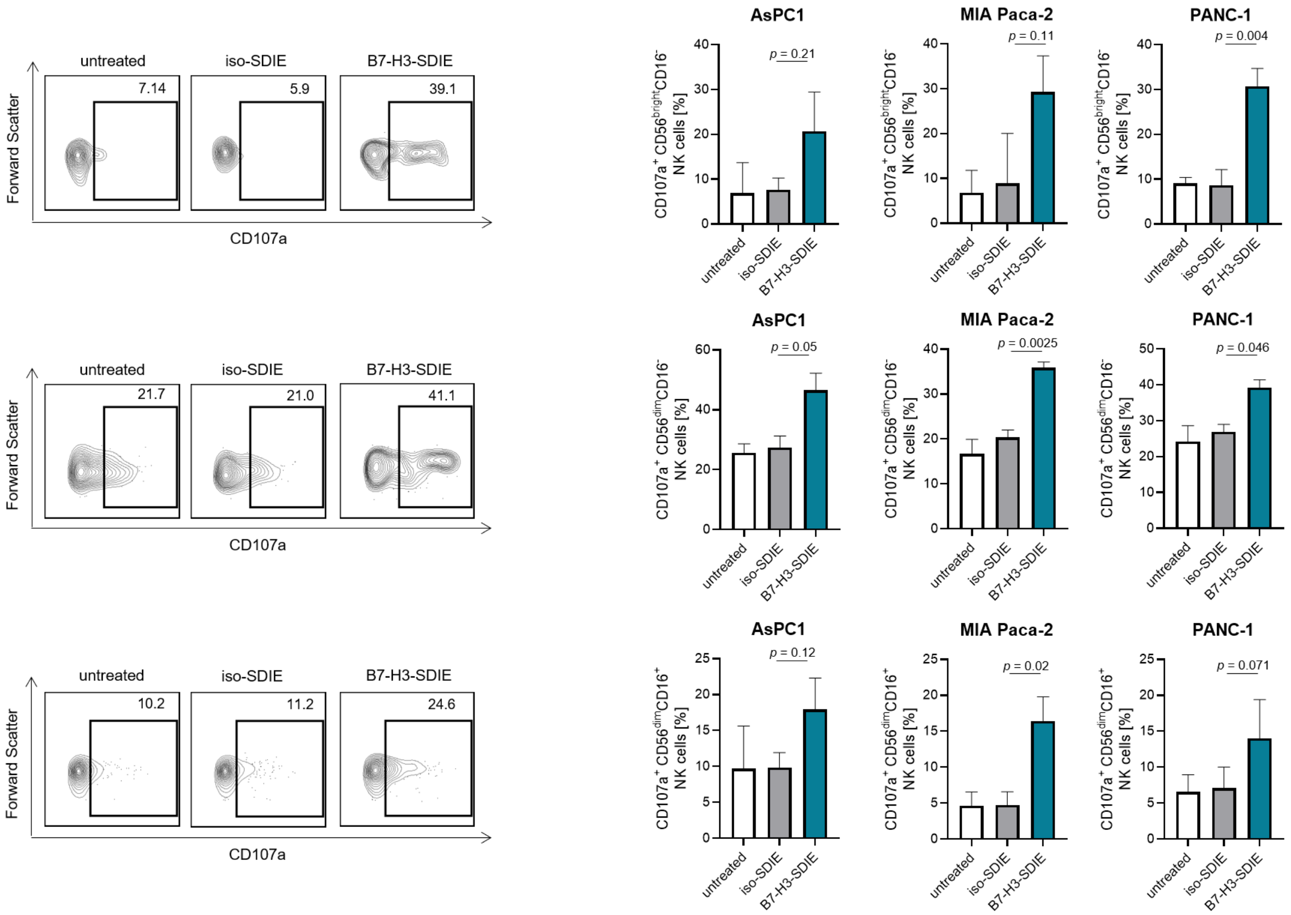

Figure S3: Degranulation of NK cell subsets (CD56^{bright}CD16^{-}, CD56^{dim}CD16^{-} and CD56^{dim}CD16^{+}) by B7-H3-SDIE. PBMC from healthy donors (n=4) were cultured with pancreatic cancer cells including AsPC-1, MIA PaCa-2, and PANC-1 at an E:T ratio of 2.5:1 with or without the B7-H3-SDIE or corresponding isotype control (1 µg/mL) followed by flow cytometry analysis. Degranulation of CD56^{bright}CD16^{-}, CD56^{dim}CD16^{-} and CD56^{dim}CD16^{+} NK cells was assessed by analyzing the expression of CD107a after 4 hours. The left panels display flow cytometry data obtained from one PBMC donor and one pancreatic cancer cell line, while the right panels exhibit results from four independent PBMC donors with pancreatic cancer cell lines. Mean ± SD is shown for the results.
